# Smoking Affects Treatment Outcome in Patients with Resected Esophageal Squamous Cell Carcinoma Who Received Chemotherapy

**DOI:** 10.1371/journal.pone.0123246

**Published:** 2015-04-13

**Authors:** Yuzhen Zheng, Xun Cao, Jing Wen, Hong Yang, Kongjia Luo, Qianwen Liu, Qingyuan Huang, Junying Chen, Jianhua Fu

**Affiliations:** 1 Department of Thoracic Oncology, Sun Yat-sen University Cancer Center; State Key Laboratory of Oncology in South China; Collaborative Innovation, Guangzhou, Guangdong, P.R. China; 2 Department of Intensive Care Unit, Sun Yat-sen University Cancer Center; State Key Laboratory of Oncology in South China; Collaborative Innovation, Guangzhou, Guangdong, P.R. China; 3 Department of Experimental Research, Sun Yat-sen University Cancer Center; State Key Laboratory of Oncology in South China; Collaborative Innovation, Guangzhou, Guangdong, P.R. China; 4 Guangdong Esophageal Cancer Institute, Guangzhou, Guangdong, P.R. China; Duke Cancer Institute, UNITED STATES

## Abstract

**Background:**

Cigarette smoking is reported to decrease survival and induce chemotherapy resistance in patients with various cancers. However, the impact of cigarette smoking on patients with esophageal squamous cell carcinoma (ESCC) remains unknown.

**Methods:**

A total of 1,084 ESCC patients were retrospectively enrolled from a southern Chinese institution. Patients were divided into two groups according to their treatment modalities: the SC group (surgery with chemotherapy) (*n* = 306) and the S group (surgery without chemotherapy) (*n* = 778). Smoking status was quantified as smoking history (non-smoker, ex-smoker, and current smoker) and cumulative smoking (0, between 0 and 20, and greater than 20 pack-years). The association between cigarette smoking and overall survival (OS) was evaluated using the Kaplan-Meier method and univariate/multivariate regression analysis.

**Results:**

Among 1,084 patients, 702 (64.8%) reported a cigarette smoking history, and the 5-year OS for non-smokers and smokers was 45.8% and 37.3%, respectively. In the SC group, compared with non-smoker, the adjusted HRs of ex-smoker and current smoker were 1.540 (95% CI, 1.1–2.2) and 2.110 (95% CI, 1.4–3.1), respectively; there is a correlative trend of decreased OS with increased cigarette smoking (*P*_trend_ = 0.001). These associations were insignificant in the S group. In subgroup analysis of the SC group, the lower OS conferred by smoking was not significantly modified by age, gender, body mass index, alcohol drinking, or chemotherapy method (chemotherapy and chemoradiotherapy).

**Conclusion:**

Our results suggest that smoking may affect treatment outcome in patients with resected ESCC who received chemotherapy.

## Introduction

Esophageal cancer is a frequent cause of death worldwide. Despite great improvements in preoperative examination, surgical technology, and multidisciplinary treatment over the past decades, the survival rate is still unsatisfactory [1–3]. Therefore, it is necessary to identify novel prognostic factors to recognize high-risk patients.

It is well documented that cigarette smoking promotes tumorigenesis [4–7]. Recently, the association between cigarette smoking and survival have been evaluated in patients with colorectal cancer [8], head and neck cancer [9], breast cancer [10], gastric cancer [11], and lung cancer [12]. However, controversy still exists regarding its role in esophageal cancer. Some studies indicated that smoking might decrease survival [13, 14], but other studies revealed no significant association [15].

Previous studies indicated that the effect of cigarette smoking on survival may be partially attributed to its interaction with chemotherapy [16–19]. Experiments in vitro also revealed that the exposure of cancer cell lines to nicotine and other metabolites of cigarette smoke can lead to chemoresistance [20–25]. However, this association is still unknown for patients with esophageal cancer.

We performed this study to evaluate the effect of cigarette smoking on long-term survival and to clarify whether it is associated with chemotherapy resistance among patients with established esophageal squamous cell carcinoma (ESCC).

## Materials and Methods

### Ethics Statement

Study approval was obtained from the independent ethics in committees at the Cancer Center of Sun Yat-sen University. Written informed consent was given by all participants for their clinical records to be used in this study. All patient data were anonymized and de-identified in a confidential manner.

### Selection of patients

Between January 2000 and December 2008, 1773 patients with pathologically confirmed ESCC were treated in the Sun Yat-sen University Cancer Center. Among these patients, 1185 (66.8%) patients who received surgical resection at the thoracic surgery department were reviewed. Excluding 69 patients without complete resection, 14 patients have distant metastases (M1), 13 patients with excessively advanced tumor status (T4b), and 5 patients with preexisting/concurrent malignant disease, 1084 patients finally constituted our study cohort. An incomplete resection included microscopically positive resection (R1), macroscopically positive resection (R2), and surgical exploration without esophagectomy. January 2000 was the date after which multidisciplinary treatment was widely applied for esophageal cancer.

Analysis of clinical stage was performed using barium esophagography, computed tomography scan of the chest, abdomen, and cervical regions, electronic gastroscopy, ultrasound gastroscopy and bronchoscopy. PET/CT was not routinely carried out. Pathologic staging was performed based on the 7th American Joint Committee on Cancer (AJCC) staging system [26]. Patients who presented pathologically complete response after neoadjuvant treatment were diagnosed as stage 0.

### Smoking status

Smoking status was translated and quantified into smoking history and cumulative smoking. In our hospital, we defined patients who had smoked more than 100 cigarettes in their lifetime as "smokers", which is similar to metrics used in previous studies [27]. Smoking history was enrolled as non-smoker, ex-smoker, or current smoker based on the medical record. Cumulative smoking was defined as pack-years (PY), which was calculated as: (the average number of cigarette smoked per day/20) × smoking years.

### Treatments

Treatment options were determined based on tumor stage, the doctor's opinion, and the patient's desires. Surgical procedure applied in this study included primary tumor resection and lymph nodes dissection. The details of these common surgical procedures are described in prior studies [3]. Chemotherapy was typically applied as a two-drug regimen of platinum-based drugs for 4–6 cycles. Preoperative radiotherapy was mainly delivered to the primary tumor with a 3 cm cranio-caudal margin, and was also applied to metastatic lymph nodes and regional lymph nodes. The total dose used was 40–50 Gy. Postoperative radiotherapy was delivered to the anastomosis, supraclavicular, and mediastinal lymphatics, with a total dose of 46–64 Gy.

### Follow-up

After primary treatment, most patients were asked to participate in outpatient follow up every three months for the first two years, every six months for years 3–5, and every 12 months thereafter. Regular assessment included physical examination, blood test, endoscopy, chest X-ray, and ultrasound test. Computed tomography scan of the chest, abdomen, and cervical region was performed at least once a year. For those who could not afford regular follow up visits, a telephone follow-up was performed instead. Survival status was reverified using the best available method in March 2014. The median time from the date of surgery to the last censoring was 68.5 months.

### Statistics

The statistical analysis was performed using the SPSS 19.0 software package (SPSS, Inc., Chicago, IL). Overall survival (OS) was defined from the date of surgery to the date of death or final follow-up. Censored cases were defined as patients (1) who were lost in contact during the follow up, and (2) who were still alive at the end of the study. The survival rate was calculated using the Kaplan-Meier method, and the differences between curves were assessed by the log-rank test. Statistical significance was assumed at a two-sided probability value < 0.05.

In this study, analysis was performed in three groups (entire cohort, patients treated by surgery with chemotherapy [SC group], and patients treated by surgery without chemotherapy [S group]). As the major exposure of interest, smoking status was analyzed in univariate analysis with other confounders, including age (as a continuous variable), gender, body mass index (BMI) (as a continuous variable), alcohol drinking (non-drinker, drinker), tumor diameter (less than or equal to 3, between 3 and 5, or greater than 5 cm), and AJCC stage (0–I, II, or III). Factors proved with statistical significance (*P* < 0.1) in univariate analysis would be introduced into multivariate analysis.

## Results

### Patient Characteristics

A total of 1084 patients were enrolled as the target population. Of these, 35.2% (382/1084) were non-smokers and 64.8% (702/1084) were smokers. The median value of cumulative smoking was 20 PY.

In this study, 63.7% (690/1084) of patients were treated by surgery alone, 8.1% (88/1084) by surgery and radiotherapy, 23.4% (254/1084) by surgery and chemotherapy, and 4.8% (52/1084) by surgery and chemoradiotherapy. Therefore, 778 patients did not received chemotherapy were divided into S group and other 306 patients into SC group. The patient characteristics are listed in Table 1.

**Table 1:** Patient clinicopathological characteristics.

Characteristics	Number (percentage)
Median age (range) (*year*)	57 (30–82)
Gender	
Male	851 (78.5)
Female	233 (21.5)
Body mass index (*kg/m* ^*2*^)	21.7±3.1
Smoking history	
Non-smoker	382 (35.2)
Smoker	702 (64.8)
Ex-smoker	411 (37.9)
Current smoker	291 (26.9)
Cumulative smoking (*pack-year*)	
0	382 (35.2)
>0&≤20	247 (22.8)
>20	455 (42.0)
Alcohol drinking	
Non-drinker	724 (66.8)
Drinker	360 (33.2)
Tumor diameter (*cm*)	
≤3	212 (19.6)
>3&≤5	462 (42.6)
>5	410 (37.8)
AJCC stage	
0-I	98 (9.0)
II	499 (46.0)
III	487 (44.9)
Treatment	
SC group	306 (28.2)
S group	778 (71.8)

SC group, surgery with chemotherapy group; S group, surgery without chemotherapy group.

### Prognostic Significance of Cigarette smoking for the Entire Cohort (n = 1084)

The 5-year OS rate was 40.0% for the entire cohort, with a median survival time of 36.0 months. In univariate analysis, we observed that smoking history (*P*_trend_ = 0.002) and cumulative smoking (*P*_trend_ = 0.001) correlated with the OS rate. After multivariate analysis, factors proven to have independent prognostic significance were smoking history, cumulative smoking, age, BMI, alcohol drinking, and AJCC stage (Table 2). The OS curves of entire cohort stratified by smoking history and cumulative smoking are presented in S1 Fig.

**Table 2:** Univariate and multivariate analysis of cigarette smoking for the entire cohort (*n* = 1084).

	Univariate analysis			Multivariate analysis					
Baseline and clinical features	uHR	95% CI	*P*-value	aHR	95% CI	*P*-value	aHR	95% CI	*P*-value
Smoking history									
Non-smoker	1			1					
Ex-smoker	1.214	1.0–1.5	0.039	1.160	1.0–1.4	0.117			
Current smoker	1.416	1.2–1.7	0.001	1.315	1.1–1.6	0.007			
*P*_trend_			0.002			0.025			
Cumulative smoking (pack-year)									
0	1						1		
>0&ati	1.131	0.9–1.4	0.251				1.176	1.0–1.5	0.135
>20	1.413	1.2–1.7	<0.001				1.382	1.2–1.7	<0.001
*P*_trend_			0.001						0.002
Age (*year*)	1.014	1.0–1.0	0.001	1.019	1.0–1.0	<0.001	1.013	1.0–1.0	0.004
Gender (male/female)	0.739	0.6–0.9	0.003	0.966	0.7–1.2	0.790	0.952	0.7–1.2	0.709
Body mass index (*kg m* ^*-2*^)	0.969	0.9–1.0	0.015	0.970	0.9–1.0	0.023	0.973	0.9–1.0	0.044
Alcohol drinking (non-drinker/drinker)	1.460	1.2–1.7	<0.001	1.348	1.1–1.6	<0.001	1.372	1.2–1.6	<0.001
Tumor diameter (*cm*) (≤3/>3&≤5/>5)	1.165	1.1–1.3	0.004	1.106	1.0–1.2	0.064	1.099	1.0–1.2	0.080
AJCC stage (0-I/II/III)	1.885	1.7–2.2	<0.001	1.919	1.7–2.2	<0.001	1.851	1.6–2.1	<0.001

uHR, unadjusted hazard ratio; CI, confidence interval; aHR, adjusted hazard ratio.

### Prognostic Significance of Cigarette Smoking for Patients with Different Treatment Modalities

**Fig 1:**
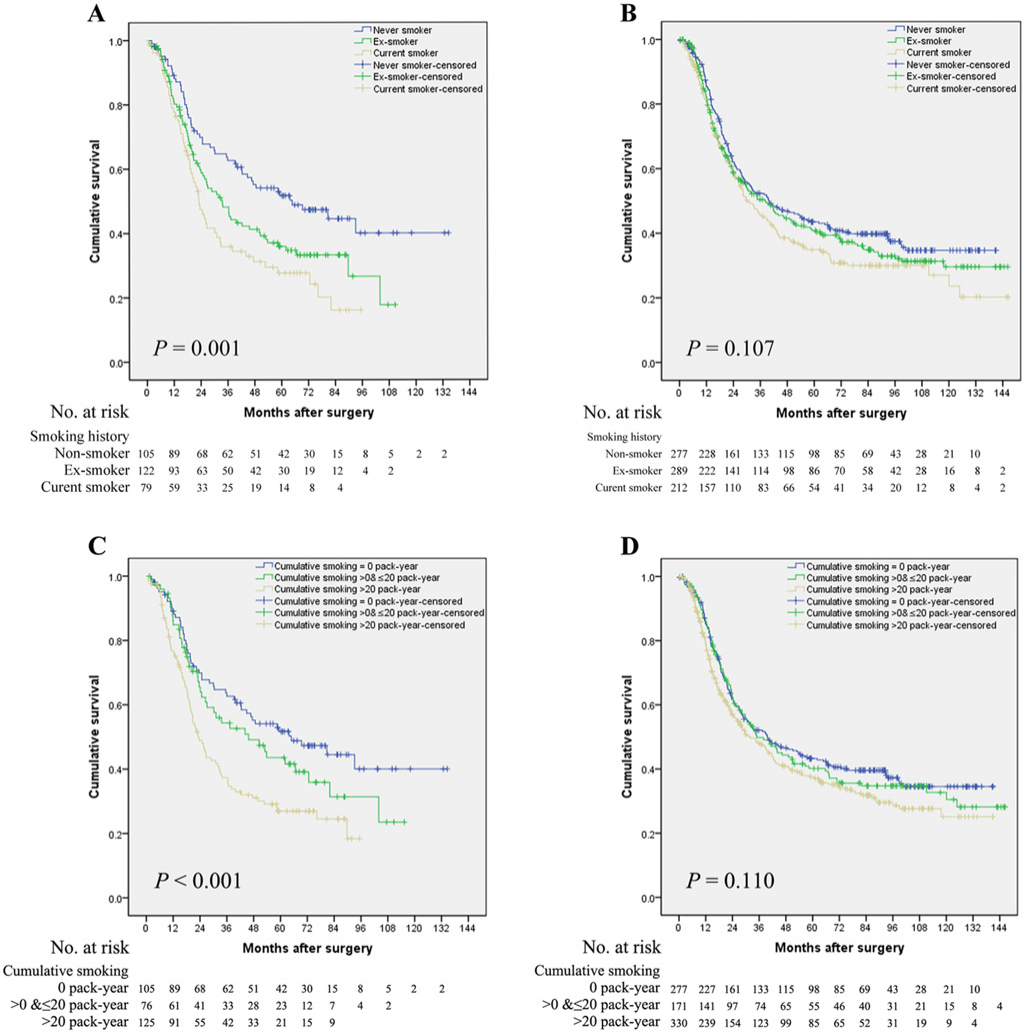
Overall survival curves stratified by cigarette smoking for esophageal squamous cell carcinoma patients with different treatment modalities. (A) In the SC group, the median survival time of non-smoker, ex-smoker, and current smoker was 64.9, 33.6, and 23.2 months, respectively (*P* = 0.001). (B) In the S group, the median survival time of non-smoker, ex-smoker, and current smoker was 39.7, 38.3, and 30.4 months, respectively (*P* = 0.107). (C) In the SC group, the median survival time of patients with cumulative smoking of 0, >0&≤20, and >20 pack-year were 64.9, 45.7, and 23.2 months, respectively (*P* < 0.001). (D) In the S group, the median survival time of patients with cumulative smoking of 0, >0&≤20, and >20 pack-year were 39.7, 34.9, and 32.1 months, respectively (*P* = 0.110). Abbreviations: SC group, surgery with chemotherapy group; S group, surgery without chemotherapy group.

In the SC group, we observed a significant association between smoking history and OS in univariate analysis (*P*_trend_ = 0.001) (Fig 1A). Compared with non-smoker, the unadjusted HR for ex-smoker and current smoker was 1.528 (95% confidence interval [CI], 1.1–2.2) and 2.014 (95% CI, 1.4–3.0). This effect was further confirmed in multivariate analysis (*P*_trend_ = 0.001); the adjusted HRs of ex-smoker and current smoker were 1.540 (95% CI, 1.1–2.2) and 2.110 (95% CI, 1.4–3.1), respectively. However, no significant association between OS and smoking history was observed in the S group (Fig 1B) (Table 3).

**Table 3:** Prognostic analysis of cigarette smoking for patients with different treatment modalities.

	Smoking history				Cumulative smoking (pack-year)						
Variable	Non-smoker	Ex-smoker	Current smoker	*P*_trend_	0	>0&≤20		>20		*P*_trend_	
In SC group (*n* = 306)											
No. of events	52	73	55		52		42		86		
No. at risk	105	121	80		105		76		125		
uHR (95% CI)	1	1.528 (1.1–2.2)	2.014 (1.4–3.0)		1		1.344 (0.9–2.0)		2.016 (1.4–2.9)		
*P*		0.020	<0.001	0.001			0.159		<0.001		<0.001
aHR (95% CI)	1	1.540 (1.1–2.2)	2.110 (1.4–3.1)		1		1.398 (0.9–2.1)		1.944 (1.4–2.8)		
*P*		0.018	<0.001	0.001			0.109		<0.001		0.001
In S group (*n* = 778) *											
No. of events	163	167	133		163		104		196		
No. at risk	277	289	212		277		171		330		
uHR (95% CI)	1	1.128 (0.9–1.4)	1.280 (1.0–1.6)		1		1.066 (0.8–1.4)		1.242 (1.0–1.5)		
*P*		0.276	0.035	0.107			0.608		0.041		0.110

SC group, surgery with chemotherapy group; S group, surgery without chemotherapy group; uHR, unadjusted hazard ratio; CI, confidence interval, aHR adjusted hazard ratio.

* Not assessed in multivariate model due to insignificant results of the univariate analysis (*P* > 0.10).

We next examined the effect of cumulative smoking on OS. In the SC group, patients with a higher consumption of tobacco had a lower OS rate in univariate analysis (*P*_trend_ < 0.001) (Fig 1C). Compared with non-smokers, the unadjusted HR for smokers with cumulative smoking of ≤20 PY and >20 PY were 1.344 (95% CI, 0.9–2.0) and 2.016 (95% CI, 1.4–2.9), respectively. In addition, the higher risk of death for patients with higher cumulative smoking remained significant in multivariate analysis (*P*_trend_ <0.001), with adjusted HRs of 1.398 (95% CI, 0.9–2.1) and 1.944 (95% CI, 1.4–2.8) for smokers with cumulative smoking of ≤20 PY and >20 PY, respectively. Nevertheless, no significant prognostic impact was observed in the S group in terms of cumulative smoking after univariate analysis (*P*_trend_ = 0.110) (Fig 1D) (Table 3).

### Subgroup Analysis for the SC group (n = 306)

The above results showed that the smoking status played an independent role in the prognosis of the SC group. We therefore broke down the SC group into subgroups stratified by baseline and clinical features to further assess the association between smoking history and OS. As shown in Table 4, the increased risk of death conferred by smoking was not significantly modified by age (≤56 and >56 years), gender, BMI (≤22 and >22 kg/m^2^), alcohol drinking, and chemotherapy method (chemotherapy and chemoradiotherapy). Additionally, this association was not significant among patients with short tumor diameter (<3 cm) and patients with early AJCC stage (0–II).

**Table 4:** Impact of cigarette smoking on overall survival based on patient characteristics for the SC group (*n* = 306).

	5-year OS rate (%)	No. of events/No. at risk		Multivariate analysis		
Factor		Non-smoker	Smoker	aHR	95% CI	*P*
Age (*year*)						
≤56*	46.9	22/48	58/106	1.620	1.0–2.5	0.045
>56	32.9	30/57	70/95	1.894	1.2–2.9	0.003
Gender						
Male	36.5	31/54	126/199	1.496	1.0–2.2	0.045
Female	56.1	21/51	2/2	4.967	1.1–22.6	0.038
Body mass index (*kg m* ^*-2*^)						
≤22.0*	37.0	27/45	71/113	1.570	1.0–2.5	0.047
>22.0	42.8	25/60	57/88	2.016	1.2–3.3	0.004
Alcohol drinking						
Non-drinker	41.4	46/92	68/112	1.487	1.0–2.2	0.039
Drinker	17.9	6/13	60/89	2.605	1.0–6.5	0.040
Tumor diameter (*cm*)						
≤3	37.0	13/25	27/41	-	-	-**
>3&≤5	39.8	25/49	43/69	1.732	1.0–2.9	0.039
>5	43.3	14/31	58/91	2.009	1.1–3.6	0.020
AJCC stage						
0-II	53.1	18/44	42/80	1.129	0.5–2.5	0.760
III	29.9	34/61	86/121	1.726	1.2–2.6	0.008
Chemotherapy method						
Chemotherapy	41.1	42/88	100/166	1.578	1.1–2.3	0.014
Chemoradiotherapy	34.8	10/17	28/35	2.683	1.3–5.7	0.011

SC group, surgery with chemotherapy group; aHR, adjusted hazard ratio; CI confidence interval.

* Median;

** Not assessed due to an insignificant result in the univariate analysis (*P* > 0.10).

## Discussion

Our study observed adverse association between cigarette smoking and long-term survival of resected ESCC. Furthermore, in subgroup analysis, the effect of cigarette smoking was restricted to patients who had received chemotherapy.

Over the past decades, the role of cigarette smoking in etiology has been well established for patients with esophageal cancer, irrespective of pathological type [4, 5]. However, little research has been done on its impact on survival. A case-control study from China observed a significantly higher esophageal cancer death rates in smokers compared with nonsmokers in all geographical groups [13]. Recently, Yaegashi et al. provided clear evidence that the outcome of esophageal cancer was adversely associated with the cumulative amount of cigarette smoking [14]. However, in another study for ESCC patients who had been treated by primary radiotherapy, there is no association between smoking status and 2-year mortality and recurrence [15]. These conflicting results were similar to our results. Although we observed significant detrimental effects of cigarette smoking in entire cohort, this association was not significant in the vast majority of patients who had been treated by surgery without chemotherapy (S group).

To our knowledge, the effect of cigarette smoking on chemotherapy has been evaluated in various human cancers [16–19]. There is only one study in esophageal cancer, and the results indicated that heavy cigarette smoking (cumulative smoking >20 PY) is a poor prognostic factor in patients with ESCC who had been treated by chemoradiotherapy [28]. However, in that study, the author combined non-smokers and light smokers into a group of non-heavy smokers (cumulative smoking ≤20 PY), which may be biased and make it difficult to clearly explain the effect of smoking behavior. Therefore, in this study, we quantified the smoking status as smoking history and cumulative smoking and proved that cigarette smoking was harmful for the entire cohort. Next, we stratified the population into two groups based on treatment modalities and observed that the detrimental impact of cigarette smoking was limited to patients who had received chemotherapy; patients with current smoking behavior and patients with higher cigarette consumption would suffer higher mortality than those with ex-smoking behavior and those with less cigarette consumption. Additionally, in subgroups based on the chemotherapy method (chemotherapy and chemoradiotherapy), this association remained unchanged after adjusting for known prognostic factors. Thus, our results strongly support that cigarette smoking would impact the treatment outcome in resected ESCC who received chemotherapy.

Although the exact mechanism for the association between cigarette smoking and chemotherapy is not known, one probable explanation would involve nicotine's interaction with the PI3K/Akt/mTOR signaling pathway [29, 30], which has been proven to participate in the regulation of chemosensitivity by its downstream effects, specifically, stimulation of angiogenesis [31] and overexpression of DNA repair enzymes [32]. Recently, several studies in vitro have demonstrated that nicotine decreased the chemosensitivity of esophageal cancer cell lines [20, 21]. Besides, presence of nicotine and carbon monoxide in the blood may also work in this process, because the smoking impact was more pronounced in current smoker in our study. Additionally, the fact that the smoking effect is remarkable in long term smokers possibly has implications for an alternate, more aggressive molecular phenotype, which may has intrinsically more chemoresistance effect, induction of detoxifying pathways, reduced oxygenation, or altered immune function.

As expected, in the subgroup analysis for SC group, we found that the increased risks associated with cigarette smoking were restricted to patients with advanced stage. A meta-analysis of ESCC observed a significantly higher OS rate among patients with stage III–IV tumors, but not in those with stage I–II tumors, with surgery plus adjuvant chemotherapy compared with surgery alone [33], which is accordance with previous studies on adjuvant chemoradiotherapy [34, 35]. Although series of studies have proven the positive prognostic impact of neoadjuvant chemoradiotherapy for esophageal cancer [2, 36], it has still failed to help those with stage I–II esophageal cancer [37]. As indicated by our results, cigarette smoking may achieve its prognostic impact by interacting with chemotherapy, therefore the mediocre effect of chemotherapy/chemoradiotherapy in patients with early stage tumors may suppress the impact of cigarette smoking. Second, cigarette smoking was not associated with survival in patients with a tumor diameter of ≤3cm. Although we cannot clearly explain the reason, this may be partially associated to the fact that 83.3% (55/66) of these patients were diagnosed as early stage (0–II).

The current study observed an adverse association between cigarette smoking and long-term survival in patients with ESCC. Therefore, control of cigarette usage should be emphasized to reduce mortality of patients of ESCC. Furthermore, our results indicated an interaction between cigarette smoking and chemotherapy for patients with ESCC. Thus, further studies should include cigarette smoking as a potential indicator for chemosensitivity and focus on the underlying mechanism.

A major limitation of our study is that we relied on self-reported smoking status and possibly mistook the exact cumulative amount of cigarette smoking. However, previous studies have demonstrated the reliability of such information [4, 16, 18, 28]. Another limitation is that we could not collect information on the age at which patients began smoking and the age at which patients stopped smoking, as these parameters might alter the effect of cigarette smoking. The third concern is of competing risks from smoking-related mortality, however, in this study, most patients died from esophageal cancer (95.2%, 610/641). We assume that if the difference in OS in the SC group were mainly due to this confounding factor, then a similar result would be seen in the S group, which has a bigger sample size.

## Conclusion

This study observed an adverse effect of cigarette smoking on the long-term survival in patients with resected ESCC who received chemotherapy. Further studies are needed to explore the biological mechanism and the effect of genetic or behavioral differences in ESCC patients regarding the presence of cigarette smoking.

## Supporting Information

S1 Fig Overall survival curves for the entire cohort stratified by cigarette smoking. (A) The median survival time of non-smoker, ex-smoker, and current smoker was 44.9, 34.7, and 28.2 months, respectively (*P* = 0.002). (B) The median survival time of patients with cumulative smoking of 0, >0&≤20, and >20 pack-year were 44.9, 40.5, and 25.5 months, respectively (*P* = 0.001).
(TIF)
