# Supplementary figures and images for: Smoking Affects Treatment Outcome in Patients with Resected Esophageal Squamous Cell Carcinoma Who Received Chemotherapy
Source: PLoS One. 2015 Apr 13;10(4):e0123246. doi: 10.1371/journal.pone.0123246 (PMC4395356; doi:10.1371/journal.pone.0123246)

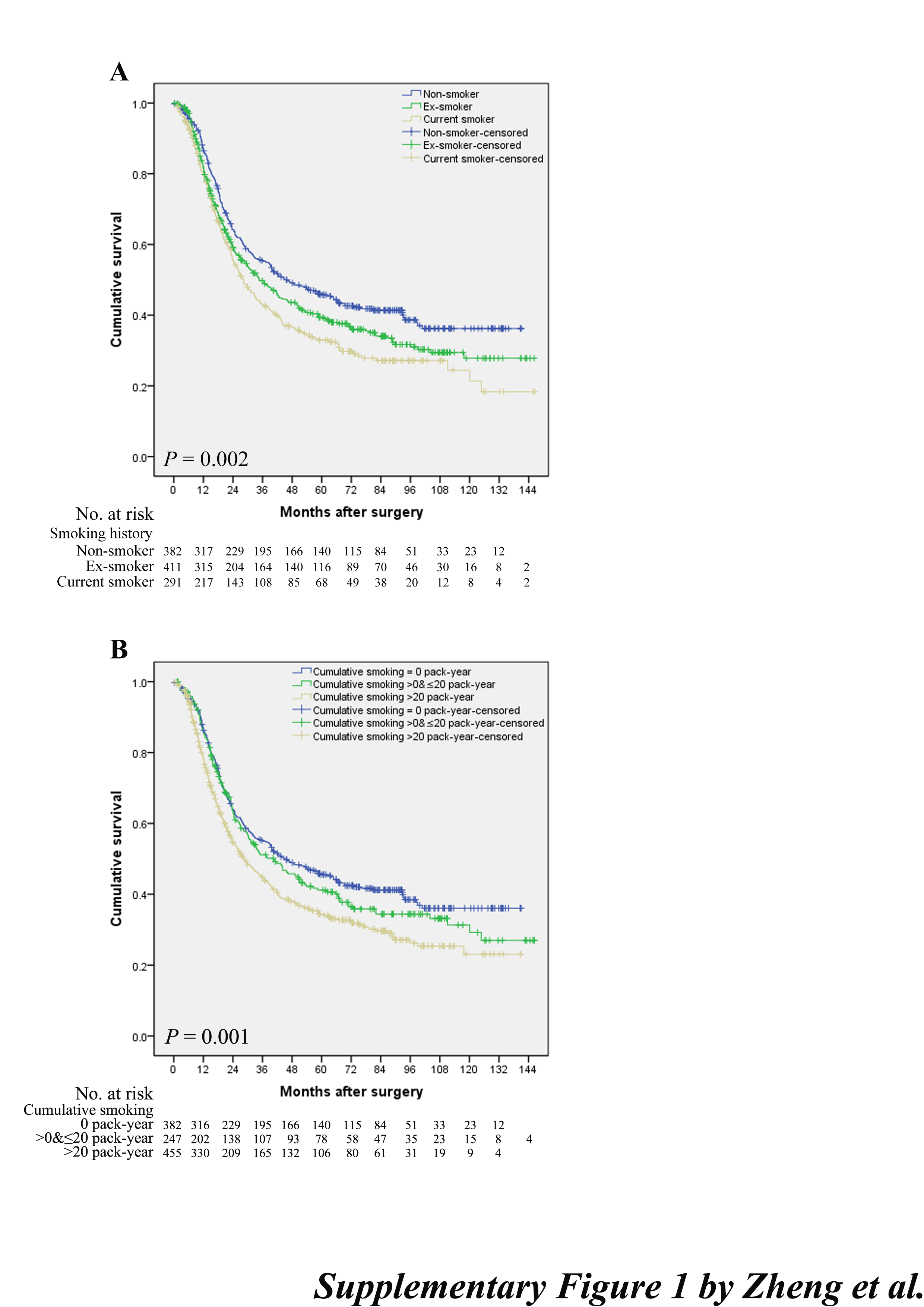

Supplement: S1 Fig — (A) The median survival time of non-smoker, ex-smoker, and current smoker was 44.9, 34.7, and 28.2 months, respectively (P = 0.002). (B) The median survival time of patients with cumulative smoking of 0, >0&≤20, and >20 pack-year were 44.9, 40.5, and 25.5 months, respectively (P = 0.001). (TIF) [file pone.0123246.s001.tif]
